# Ocean acidification exerts negative effects during warming conditions in a developing Antarctic fish

**DOI:** 10.1093/conphys/cov033

**Published:** 2015-07-27

**Authors:** Erin E Flynn, Brittany E Bjelde, Nathan A Miller, Anne E Todgham

**Affiliations:** af1 Department of Biology, San Francisco State University, San Francisco, CA 94132, USA; af2 Department of Animal Sciences, University of California, Davis, CA 95616, USA

**Keywords:** Early development, global climate change, *Gymnodraco acuticeps*, physiological performance, polar fishes

## Abstract

Anthropogenic CO_2_ is rapidly causing oceans to become warmer and more acidic, challenging marine ectotherms to respond to simultaneous changes in their environment. While recent work has highlighted that marine fishes, particularly during early development, can be vulnerable to ocean acidification, we lack an understanding of how life-history strategies, ecosystems and concurrent ocean warming interplay with interspecific susceptibility. To address the effects of multiple ocean changes on cold-adapted, slowly developing fishes, we investigated the interactive effects of elevated partial pressure of carbon dioxide (*p*CO_2_) and temperature on the embryonic physiology of an Antarctic dragonfish (*Gymnodraco acuticeps*), with protracted embryogenesis (∼10 months). Using an integrative, experimental approach, our research examined the impacts of near-future warming [−1 (ambient) and 2°C (+3°C)] and ocean acidification [420 (ambient), 650 (moderate) and 1000 μatm *p*CO_2_ (high)] on survival, development and metabolic processes over the course of 3 weeks in early development. In the presence of increased *p*CO_2_ alone, embryonic mortality did not increase, with greatest overall survival at the highest *p*CO_2_. Furthermore, embryos were significantly more likely to be at a later developmental stage at high *p*CO_2_ by 3 weeks relative to ambient *p*CO_2_. However, in combined warming and ocean acidification scenarios, dragonfish embryos experienced a dose-dependent, synergistic decrease in survival and developed more slowly. We also found significant interactions between temperature, *p*CO_2_ and time in aerobic enzyme activity (citrate synthase). Increased temperature alone increased whole-organism metabolic rate (O_2_ consumption) and developmental rate and slightly decreased osmolality at the cost of increased mortality. Our findings suggest that developing dragonfish are more sensitive to ocean warming and may experience negative physiological effects of ocean acidification only in the presence of an increased temperature. In addition to reduced hatching success, alterations in development and metabolism due to ocean warming and acidification could have negative ecological consequences owing to changes in phenology (i.e. early hatching) in the highly seasonal Antarctic ecosystem.

## Teaser

Polar areas are experiencing some of the most rapid impacts of climate change, yet we have a limited understanding of biological vulnerability to multiple stressors. Increased temperature broadly affected early embryo physiology in the naked dragonfish, while ocean acidification interacted synergistically with temperature to decrease survival and alter developmental rate.

## Introduction

Rapid changes to our oceans induced by anthropogenic carbon dioxide emissions have the potential to alter oceanic life fundamentally (Doney *et al*., 2012). A global average increase in temperature of ∼1°C has already led to altered geographical distribution of species, with marine ectotherms maintaining thermal environments through poleward expansion and equatorial contraction (Sunday *et al*., 2012). The concurrent absorption of ∼25% of emitted CO_2_ by the world's oceans is altering the seawater chemistry by reducing pH and changing the balance of carbon species (e.g. carbonate and bicarbonate; Ciais *et al*., 2014). These effects, termed ocean acidification, have emerged as a separate consequence of increased carbon emissions with potentially negative, broad-ranging effects on marine species and ecosystems (Orr *et al*., 2005; Hofmann and Todgham, 2010; Kroeker *et al*., 2010). Inquiry into this nascent field has increased over the past decade, but there is a pressing need to fill current gaps in our understanding of the impact of multiple, simultaneous, climate change-related stressors on marine organisms (Todgham and Stillman, 2013). In particular, we have a very limited understanding of the effects of multiple stressors on organisms living in vulnerable ecosystems and under-researched organisms at sensitive life stages (Wernberg *et al*., 2012).

While it is possible to predict future scenarios of temperature increase and ocean acidification based on CO_2_ and other greenhouse gas emissions (IPCC, 2013), estimating the biological impacts of simultaneously changing abiotic conditions proves much more complex (Crain *et al*., 2008; Darling and Côté, 2008; Holmstrup *et al*., 2010). In some instances, the combination of two abiotic perturbations is simply the sum of each individual change (additive); however, many times we cannot easily predict when more complicated, non-linear interactions may occur, such as when combined effects are smaller (antagonistic) or greater than their sum (synergistic). For example, in developing marine invertebrates exposed to factorial combinations of increased temperature and partial pressure of carbon dioxide (*p*CO_2_), there are most often negative additive or antagonistic effects at the organismal level, and synergistic interactions occur only on occasion (Byrne and Przeslawski, 2013).

Despite their strong capacity to acid–base regulate, marine fishes have shown vulnerability to ocean acidification (Ishimatsu *et al*., 2008; Munday *et al*., 2010; Baumann *et al*., 2011; Frommel *et al*., 2011; Esbaugh *et al*., 2012; Bignami *et al*., 2013; Enzor *et al*., 2013; Hurst *et al*., 2013). Additionally, temperature is a primary abiotic driver of fish physiology and ecology (e.g. Beitinger and Fitzpatrick, 1979; Houde, 1989). Ocean warming is predicted to have broad-reaching impacts on marine fishes (Rijnsdorp *et al*., 2009; Pörtner and Peck, 2010), including already documented poleward expansions (Perry *et al*., 2005; Figueira and Booth, 2010) and reduced growth efficiencies at lower latitudes (Neuheimer and Grønkjær, 2012). To date, research on the effects of simultaneous warming and ocean acidification on marine fishes has largely focused on adults (Munday *et al*., 2009a; Strobel *et al*., 2012; Enzor *et al*., 2013), but early life stages may be most vulnerable (Pankhurst and Munday, 2011). While fish embryos generally possess a narrow temperature tolerance window and commonly exhibit increased mortality, growth and development rate during warming (Rombough, 1997), early life stages of fishes exhibit highly variable interspecific responses to ocean acidification. Some studies have found that ocean acidification reduces hatching success or survival (Baumann *et al*., 2011; Forsgren *et al*., 2013; Chambers *et al*., 2014), while other studies show no changes in mortality (Munday *et al*., 2009b; Franke and Clemmesen, 2011; Frommel *et al*., 2013; Hurst *et al*., 2013). From the limited number of studies that have exposed fish embryos to both warming and ocean acidification, later larval stages experienced either no interactive effects (Frommel *et al*., 2013) or multiple interactive effects (Pimentel *et al*., 2014b) in the presence of both stressors. Currently, there are insufficient studies that have investigated the effects of multiple changes in ocean conditions on marine organisms to draw generalities or species-specific patterns. No studies have been conducted on effects of either increased *p*CO_2_ or temperature on developing Antarctic fishes, which are predicted to have limited abilities to cope with thermal stress due to their long evolution at sub-zero temperatures (Coppes Petricorena and Somero, 2007) and may likewise have limited capacity to acclimatize to rapid changes in *p*CO_2_ predicted to occur in Antarctic waters within the next century (McNeil and Matear, 2008; McNeil *et al*., 2010).

Polar organisms, living in the coldest marine ecosystems, are vulnerable to climate change specifically because of their adaptation to historically stable thermal regimens, the rapid speed at which the poles are currently changing, and their slow population growth (Meredith and King, 2005; Smetacek and Nicol, 2005; Barnes and Peck, 2008; Barnes *et al*., 2009). Compared with fishes in other ecosystems, the range of temperatures over which Antarctic ectotherms can maintain physiological function is one of the smallest, and adult fishes are thought to be able to acclimatize to conditions only 4.5°C warmer than their current mean maximal temperature (Somero and DeVries, 1967; Podrabsky and Somero, 2006; Richard *et al*., 2012). Cold polar waters are also able to absorb more CO_2_ than warmer waters, making the Southern Ocean a prominent CO_2_ sink and a potential ‘bellwether’ of the effects of ocean acidification to marine life, which are expected to reach conditions unfavourable for calcifying organisms as soon as 2050 (McNeil and Matear, 2008; Fabry *et al*., 2009; McNeil *et al*., 2010). As high-latitude Antarctic species are limited in their ability to relocate to favourable conditions, the primary strategies for coping with rapid environmental change rely on possessing the flexibility to acclimatize to their new environment through shifts in physiology (i.e. energy allocation, stress response), behaviour (i.e. foraging rate, microhabitat usage) and/or gene pool (i.e. population-level increase in better-performing genotypes; Pörtner and Farrell, 2008). While the effects of ocean acidification on Antarctic animals that use calcium carbonate to form their shells is receiving increasing attention [e.g. pteropods (Bednaršek *et al*., 2012), sea urchins (Sewell and Hofmann, 2011; Byrne *et al*., 2013)], research on non-calcifying Antarctic marine species, especially in conjunction with warming temperatures, is limited.

Notothenioid fishes, the most specious and abundant (by biomass) Antarctic fish suborder (Eastman, 2000), have limited ability to cope with warm temperature stress (Somero and DeVries, 1967; although see Franklin *et al*., 2007; Bilyk and DeVries, 2011) after millions of years of evolution at sub-zero temperatures and are widely believed to be very sensitive to ocean climate change (Somero, 2010; Patarnello *et al*., 2011; Mintenbeck *et al*., 2012; O'Brien and Crockett, 2013). In adult Antarctic notothenioid species exposed to temperature and hypercapnic stress, routine metabolic rate tends to remain high after temperature acclimation, but some species can acclimate to hypercapnic stress over time (Strobel *et al*., 2012; Enzor *et al*., 2013). However, higher *p*CO_2_ levels appear to alter cellular processes, particularly involving mitochondrial respiration, although effects vary by tissue and interaction with temperature (Strobel *et al*., 2013a,b). While these studies pave the way for understanding how a changing Antarctic environment will affect adult fish, we have no information about the sensitivities of the earliest life stages, embryos and larvae, which are speculated to be even more vulnerable to these multiple stressors (Mintenbeck *et al*., 2012).

To investigate the sensitivity of developing Antarctic fishes to ocean climate change, we assessed the effects of near-future ocean warming and acidification on early embryos of the naked dragonfish, *Gymnodraco acuticeps* (Boulenger 1902). *Gymnodraco acuticeps* is a shallow benthic spawner with protracted embryogenesis (∼10 months) exclusive to the circumpolar Antarctic (Evans *et al*., 2005), and thus embryos may be especially vulnerable to altered abiotic conditions due to habitat and life history. As the first study to investigate the response to warming and increased *p*CO_2_ in a developing Antarctic fish, we designed our experiment to capture both lethal and sub-lethal physiological changes over a short period (i.e. 3 weeks) in early development, because early embryogenesis has previously been shown to be a highly sensitive stage to increased *p*CO_2_ (Kikkawa *et al*., 2003; Forsgren *et al*., 2013) and temperature (Rombough, 1997). Based on previous multistressor research, we predicted that tolerance to a single stressor (i.e. increased *p*CO_2_) would be altered during concurrent exposure to a second stressor (Todgham and Stillman, 2013), likely in a negatively additive fashion (Byrne and Przeslawski, 2013). Survival over time was measured, because any changes in future ocean conditions during embryogenesis could have significant effects on hatching success and ultimately recruitment and population growth. Likewise, we also focused on measures of sub-lethal stress that could have potential negative consequences on future fitness: development and metabolism. Changes to developmental progression, particularly more rapid development, could affect the timing of hatching in the extremely seasonal Antarctic environment and lead to a phenological mismatch between fish larvae emergence and prey availability (Edwards and Richardson, 2004). As alterations to development could be the result of increased or decreased energy demand or changes in the amount of energy put towards growth vs. maintaining homeostasis in unfavourable biological conditions (e.g. the oxygen and capacity limited tolerance hypothesis of Pörtner, 2012; including a bioenergetic framework described by Sokolova *et al*., 2012), we also measured embryo metabolism. By measuring metabolic processes at the whole-organism (O_2_ consumption) and cellular levels (aerobic enzyme activity), we sought to disentangle metabolic rate from development and broadly determine whether changes in the environment required more energy for other cellular processes, such as osmoregulation.

## Materials and methods

### Study species

The naked dragonfish, *Gymnodraco acuticeps* (Boulenger 1902) is a benthic dwelling Antarctic dragonfish (family Bathydraconidae) of the notothenioid suborder found exclusively in the circumpolar Antarctic, and adults (up to 35 cm) feed primarily on fish and invertebrates (La Mesa *et al*., 2004). Annual spawning in McMurdo Sound in the Ross Sea occurs from mid-October to early November within the shallow shelf (<50 m) on flat rocks, and larval fish hatch the following year in late August to early September (Evans *et al*., 2005). Nest guarding has been observed by both parents at different time intervals and includes behaviours such as egg fanning and aggression towards intruders (Evans *et al*., 2005). In the Ross Sea, fish such as the Antarctic toothfish (*Dissostichus mawsoni*) and *Trematomus newsii* are potential predators of the dragonfish (La Mesa *et al*., 2004), while notothenioid eggs in general serve as prey for a diverse range of animals, such as fishes, seals and invertebrates (Kock and Kellermann, 1991). Dragonfish embryos have one of the longest developmental durations in teleost fish, which may involve periods of metabolic quiescence during dark winter months (Evans *et al*., 2005, 2006).

### Collection of embryos

Antarctic dragonfish (*G. acuticeps*) egg masses were first detected on 14 October 2013 at the water intake jetty in front of McMurdo Station on Ross Island, Antarctica (77°51′4.04″S, 166°39′55.45″E) in McMurdo Sound by SCUBA divers during the first dive of the summer season. Eggs were subsequently collected on 8 November 2013, from one area of two patches suspected to be from the same female of the same lay date (estimated to be approximately 1 November based on embryo ageing in Evans *et al*. 2005). Following collection, eggs were returned to the A.P. Crary Science and Engineering Center at McMurdo Station, where they were held in a flow-through seawater table at −1 to −0.5°C (ambient incoming seawater temperature) under ambient light for 1 week prior to experimentation. Experimental procedures, handling and care were reviewed and approved by the San Francisco State Institutional Animal Care and Use Committee (protocol no. A10-005).

### Experimental carbon dioxide system “and acclimation

Average seawater temperature in the Ross Sea is −1.8°C, with seasonal warming in the upper 200 m reaching −1.7 to +0.5°C (Cziko *et al*., 2006), and spring (October to November) pH mean values at 15 m depth range from 8.02 to 8.05 depending on location (Hofmann *et al*., 2011). While longer-term monitoring has recently revealed that annual summer productivity is associated with a seasonal increase in pH (Kapsenberg *et al*., 2015), we used experimental pH values and future predictions based on values relevant for the spring developmental period of the study species. In future climate scenarios, within the next 85 years the sea surface temperature is predicted to warm by +3°C, and oceanic *p*CO_2_ levels could reach 1000 μatm, with pH dropping to 7.6 (RCP8.5 scenario, IPCC, 2013).

To assess potential vulnerability of developing dragonfish to future ocean scenarios, we created a fully factorial experimental design with two temperatures [ambient (−1°C, low) and +3°C increase (+2°C, elevated)] and three *p*CO_2_ levels [ambient (420 μatm), moderate (650 μatm) and high (1000 μatm)]. Two different temperature treatments were maintained by splitting incoming seawater into 680 l tanks at either a greater [−0.78 ± 0.09°C (mean tank temperature ± SD)] or lower flow rate (1.7 ± 0.2°C) that held 19 l square reservoir and culture buckets. For each temperature level, there were three replicate culture buckets for each of the three *p*CO_2_ treatments. Seawater chemistry was manipulated according to modified methods of Fangue *et al*. (2010) by using mass flow valves (Sierra Instruments, Monterey, CA, USA) to mix pure CO_2_ gas and ambient air stripped of CO_2_ and moisture to achieve the desired *p*CO_2_ levels. The resulting gas mixture was bubbled into seawater reservoir buckets (one for each temperature × *p*CO_2_ level) using venturi injectors, and the equilibrated treatment seawater was dripped into three replicate culture buckets at 16 l h^−1^ to maintain high water turnover. Additionally, the same gas mixture was bubbled directly into culture buckets using air stones to provide a high level of mixing within the buckets.

Two days before the start of the experiment, egg masses were gently separated into individual embryos, non-viable embryos were removed, and embryos were sorted randomly into floating mesh baskets (27 per basket × 3 = 81 embryos per culture bucket). Mesh baskets consisted of three plastic reusable coffee filters glued together with a sealed airline tubing float that kept the negatively buoyant eggs fully submerged in their treatment conditions. Experimental start times were staggered by 1 day between each temperature treatment. Embryos in the elevated temperature group were first transferred to +1°C for 24 h before being transferred to experimental tanks at +2°C across three *p*CO_2_ levels. Embryos were acclimated for up to 3 weeks in experimental conditions, with a subset of embryos sampled following 24 h and 1, 2 and 3 weeks of acclimation. An acclimation of up to 3 weeks allowed us to examine both the timing and the mechanisms of response without prior information about tolerance to elevated temperature or *p*CO_2_.

### Seawater chemistry

Temperature was measured every day in culture buckets using a hand-held thermocouple thermometer (HH81A; Omega, Stamford, CT, USA), and tank temperature was additionally recorded using temperature loggers every 30 min (Onset HOBO Data Loggers, Bourne, MA, USA). Total pH was measured every other day spectrophotometrically (UV Spectrophotometer; Shimadzu, Columbia, MD, USA) using *m*-cresol dye (Sigma-Aldrich, St Louis, MO, USA; Dickson *et al*., 2007). Total alkalinity was measured every 4 days using open-cell titration (T50 titrator; Mettler-Toledo Inc., Columbus, OH, USA; titrant and reference standards from Dickson Laboratory, Scripps Institute, La Jolla, CA, USA; Dickson *et al*., 2007). Experimental *p*CO_2_ values were calculated from total pH, *in situ* temperature, alkalinity and salinity using the package seacarb (v2.4.10; Lavigne and Gattuso, 2013) in R (R Development Core Team, 2013). Seawater chemistry over the course of the experiment is summarized in Table 1.

**Table 1:** Seawater chemistry of experimental treatments

Treatment	Temperature (°C)	pH (total scale)	Alkalinity (μmol kg^−1^)	*p*CO_2_ (μatm)	Salinity
Incoming seawater	−1.0 ± 0.2	7.962 ± 0.005	2350.7 ± 3.5	484 ± 6	33.7 ± 0.3
Temperature −1°C					
Ambient CO_2_	−0.6 ± 0.2	8.01 ± 0.01	2351.8 ± 1.9	425 ± 9	33.8 ± 0.3
Moderate CO_2_	−0.6 ± 0.2	7.84 ± 0.02	2350.6 ± 2.6	658 ± 28	33.7 ± 0.3
High CO_2_	−0.6 ± 0.1	7.66 ± 0.01	2350.9 ± 2.5	1008 ± 31	33.8 ± 0.4
Temperature +2°C					
Ambient CO_2_	1.9 ± 0.2	7.99 ± 0.01	2351.9 ± 1.7	456 ± 11	33.7 ± 0.3
Moderate CO_2_	2.0 ± 0.2	7.84 ± 0.01	2350.9 ± 1.8	669 ± 18	33.7 ± 0.3
High CO_2_	2.0 ± 0.2	7.70 ± 0.01	2351.8 ± 2.2	935 ± 24	33.7 ± 0.3

Temperature, pH, alkalinity and salinity were measured *in situ*, and partial pressure of carbon dioxide (*p*CO_2_) was calculated using seacarb (Lavigne and Gattuso, 2013) in R (R Development Core Team, 2013). All values are means ± SD.

### Survival

Survival was assessed at the 1, 2 and 3 week time points by visual inspection and gentle manipulation during sampling to minimize disturbance stress. Samples were considered non-viable and removed if they contained no embryo or yolk, the embryo became white and asymmetrical, or the external egg contained a white mass, indicating that the egg had become infected or that the internal egg milieu was breached.

### Respirometry

Whole-organism respiration rate was determined at 1, 2 and 3 week time points by measuring the rate of oxygen consumption in a closed respirometry system similar to that of Evans *et al*. (2006). Although not a standard approach, mechanical shaking was not incorporated owing to the sensitivity of early embryonic stages to shaking. A previous study on Antarctic fish embryos using a similar respirometer set-up found that mixing did not change embryonic oxygen consumption rates (Evans *et al*., 2012). Oxygen saturation was measured using external fibre-optic probes coupled with oxygen-sensor spots affixed to the inside of glass respirometry chambers (Witrox 4; Loligo Systems, Tjele, Denmark). The system was calibrated before each time point using 1% sodium sulfite (0% O_2_ saturation) and fully O_2_-saturated seawater (100% air saturation). Due to the low rate of O_2_ consumption, groups of five embryos were pooled either from the same culture bucket or from the same treatment to ensure robust detection of respiration (one replicate from each bucket and two replicates pooled across buckets for *n* = 5 per *p*CO_2_ × temperature × time). Eggs were placed into 1.2 ml respirometry chambers filled with fully O_2_-saturated water from their respective *p*CO_2_ and temperature treatments without bubbles. All respirometry measurements occurred in the dark at treatment temperature (−1.2 ± 0.1°C low or 2.0 ± 0.2°C high temperature), and percentage O_2_ saturation was measured for 3 h using Loligo software. Oxygen saturation never dropped below 80% total saturation, and at least one blank for each *p*CO_2_ treatment was run per sampling time point per temperature to account for background biological activity in the seawater. Following the respirometry trial, all eggs were assessed for viability.

To calculate respiration rate, the percentage O_2_ saturation was converted into moles of O_2_ per litre, and a linear regression model was fitted to calculate the change in O_2_ concentration over time. The first 60 min of recordings were discarded to remove any influence of handling stress on embryos and to allow sensor spots to cool to temperature. Preliminary analysis demonstrated that metabolic rate stabilized after 60 min of embryos being placed in the respirometer. Respiration rate (expressed as nanomoles of O_2_ per individual per hour) was calculated based on the number of viable embryos per vial and the volume of the respirometry chambers (1.2 ml) minus the volume of eggs (average diameter = 3.35 mm, assuming spherical shape). All respiration rates were corrected for the background rate of the blanks (*n* = 2–4 per temperature at each time point).

### Morphometrics and development

After each respirometry trial, embryos were transferred to 30 ml scintillation vials containing fully oxygenated treatment water on ice, and photographed in an environmental room (−1°C) to assess development and growth. A stereoscope fitted with a digital camera (scope from Wild Heerbrugg; adapter from Carl Zeiss; and camera, Canon Power Shot A630) was used to take a group photograph and individual photographs of each egg using a 0.01 mm micrometer for reference. Three embryos from each replicate bucket were blotted dry and weighed to the nearest 0.01 mg.

**Figure 1:**
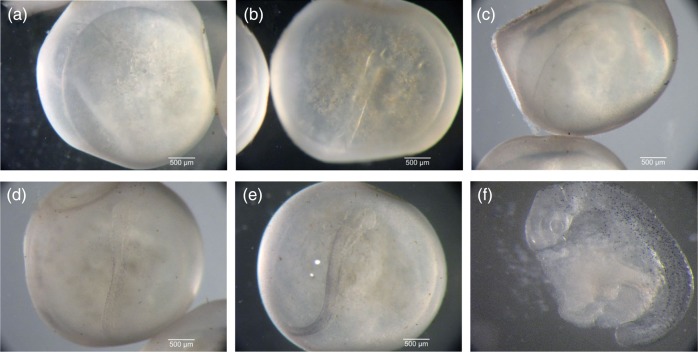
Photographs of dragonfish (*Gymnodraco acuticeps*) embryonic development stages during experiment. (**a**) Embryo undergoing gastrulation. (**b**) Early segmentation with no visible pigmentation, translucent appearance (pigment level = 0). (**c**) Primitive eyes become visible during early segmentation. (**d**) Onset of pigmentation during segmentation with diffuse, spotty pigments forming along outer edge of notochord (pigment level = 1). (**e**) Continued segmentation, with distinct lines of pigment along notochord, somite pigments visible, especially towards tail, and embryo is visible through chorion without magnification (pigment level = 2). (**f**) Embryo out of chorion during somitogenesis.

All images were analysed blindly (without knowledge of treatment) in ImageJ (v10.2) and calibrated to micrometer photographs. Egg diameter was calculated as the mean of two perpendicular diameters of the egg exterior (*n* = 25 per *p*CO_2_ × temperature × time). Approximate ageing was determined from previous work on dragonfish during fertilization through epiboly (Evans *et al*., 2005) and visual comparisons with development of a sub-polar notothenioid, *Patagonotothen ramsayi* (Arkhipkin *et al*., 2013). During somitogenesis, embryos begin to develop the melanophores that give them their distinctive larval spotting pattern, which allowed qualitative ageing of dragonfish embryos within their semi-translucent chorions (Ahlstrom and Moser, 1980). Embryos at 2–3 weeks were assigned an age ranking from 0 to 2 based on the following criteria: 0 = no visible pigmentation, translucent (Fig. 1b and c); 1 = diffuse, spotty pigmentation forming along outer edge of notochord (Fig. 1d); and 2 = distinct lines of pigment along notochord, pigmented somites visible, especially towards the tail, embryo visible through chorion without magnification (Fig. 1e). Embryos were excluded from analysis if embryo stage could not be assigned accurately due to the embryo position or poor image quality, thus *n* = 13–25 per *p*CO_2_ × temperature × time.

### Biochemical assays

#### Sample collection

Viable embryos were carefully removed from mesh baskets at 24 h, 1, 2 and 3 week time points, gently blotted dry, placed in cryovials and immediately flash frozen in liquid nitrogen. All samples were stored at −80°C until biochemical analyses.

#### Citrate synthase

Citrate synthase (CS) enzyme activity, a measurement of cellular aerobic potential in optimal conditions, was quantified in embryos (*n* = 9 per *p*CO_2_ × temperature × time) following the methods of Jayasundara *et al*. (2013) modified for a 96-well microplate. Individual embryos were homogenized in 100 μl of ice-cold 50 mM potassium phosphate buffer (pH 6.8 at 20°C) in 0.7 ml glass Dounce homogenizers on ice, which allowed the egg chorion to be separated and removed. Samples were centrifuged at 1000***g*** for 10 min at 4°C to pellet external egg sediment, large pieces of the chorion and cellular debris. The supernatant was transferred to a new microcentrifuge tube, and 10 μl of sample was loaded in sextuplicate onto a Costar clear polystyrene plate on ice. Citrate synthase buffer contained 50 mM imidazole (pH 8.2), 1.5 mM MgCl_2_, 0.1 mM Ellman's reagent [5,5′-dithio*bis*-(2-nitrobenzoic acid) or DTNB] and 0.12 mM acetyl CoA. To one set of triplicates, 200 μl of citrate synthase buffer containing 0.5 mM oxalacetic acid was added, while the other triplicate set received 200 μl of citrate synthase buffer without the substrate, to measure background activity. Enzyme activity was monitored in a plate reader (Biotek Synergy HT, Winooski, VT, USA) at 412 nm at 25°C for 2 h using a kinetic sweep and the Biotek Gen5 software to calculate the maximal rate of change in absorbance. The first 10 min of data were not used for analysis to allow the samples to temperature equilibrate, and the maximal CS enzyme activity was calculated from 20 consecutive points for the best linear fit. Citrate synthase activity was calculated by subtracting the mean background activity for each sample and converting to picomoles of substrate converted per minute per egg using the molar extinction coefficient of DTNB (14.1 ml μmol^−1^ cm^−1^) and an optical path length of 0.59 cm. Ten microlitres of embryo homogenate was stored at −20°C and later analysed for total protein concentration using the bicinchoninic acid assay with bovine serum albumin as a protein standard (Smith *et al*., 1985; Thermo Fisher Scientific, Rockford, IL, USA).

#### Osmolality

Individual eggs were diluted 3.5 times (w:v) in Millipure water, sonicated, and centrifuged at 14 000***g*** for 10 min at 4°C. Clear supernatant was collected and stored on ice until osmolality analysis. Osmolality was measured in triplicate for each sample using a vapour pressure osmometer (5600 Vapro; Wescor, Logan, UT, USA) calibrated with 100, 290 and 1000 mosmol kg^−1^ standards using a 2 μl sample volume (*n* = 9, except for 3 weeks at elevated temperature [ambient (*n* = 8), moderate (*n* = 5) and high (not analysed)] owing to mortality.

### Statistical methods

All statistical analyses were conducted using R (R Development Core Team, 2013) with the Rstudio user interface (v 0.98.836). The α level was set at *P *<* *0.05 for all analyses.

#### Survival analysis

Survival curves were compared between temperature and *p*CO_2_ levels using the *Survival* (v2.37; Therneau, 2013) and *Interval* (Fay and Shaw, 2010) packages in R to allow for interval mortality assessment and right-censored data due to sampling. Differences between each temperature and *p*CO_2_ treatment combination curve were assessed using the permutation form of the asymptotic logrank k-sample test, and differences between individual curves were determined by Sun's score statistics, where positive values indicate earlier failure than expected.

#### Developmental staging

Pearson's χ^2^ tests were used to assess the effects of treatment on development based on count data using Monte Carlo methods to estimate *P*-values based on 2000 simulations. The effects of temperature were assessed by comparing the following factors: (i) low temperature 2 weeks vs. high temperature 2 weeks; (ii) low temperature 3 weeks vs. high temperature 3 weeks; and (iii) low temperature 3 weeks vs. high temperature 2 weeks. The effects of *p*CO_2_ were then tested within each temperature and time treatment using the Bonferroni correction.

#### Oxygen consumption, egg mass, egg length, citrate synthase enzyme activity and osmolality

Remaining metrics were analysed using analysis of variance (ANOVA), with temperature, *p*CO_2_ and time as fixed factors and with culture bucket replicate included as a random effect when applicable (significance determined by restricted maximum likelihood <0.05, adjusted for testing on the boundary). Normality and homogeneity of residuals were validated through visual inspection of qq plots, fitted values vs. residuals, and factor levels vs. residuals. Significant heterogeneity within factor levels was incorporated into a generalized least-squares model using the ‘varIdent’ variance structure (Zuur *et al*., 2009). Significance of fixed effects and their interactions was assessed using the *anova* function on linear, linear-mixed effects or generalized least-squares models in the *car* (Fox and Weisberg, 2010) or *nlme* package (Pinheiro *et al*., 2014), and followed by Tukey's *post hoc* tests to determine differences among treatments [*multcomp* (Hothorn *et al*., 2008) or Tukey's honest significant difference].

## Results

### Survival

**Figure 2:**
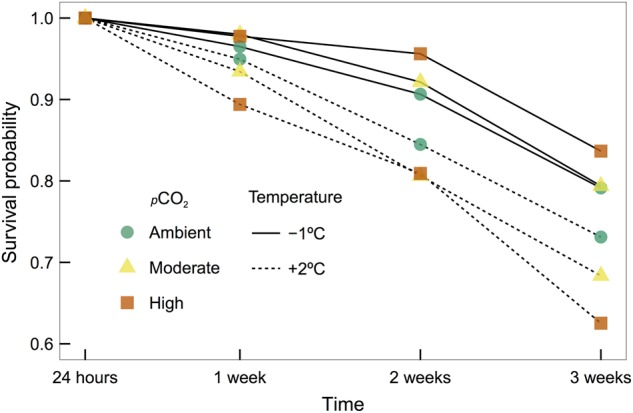
Cumulative survival probability of dragonfish (*G. acuticeps*) embryos over the course of 3 weeks with acclimation to either −1 or +2°C and ambient (420 μatm), moderate (650 μatm) or high (1000 μatm) partial pressure of carbon dioxide (*p*CO_2_). Survival was assessed at 1, 2 and 3 weeks, and individuals sampled at each time point were considered right censored.

Temperature and *p*CO_2_ interactively affected survival (non-parametric log-rank test, χ^2^ = 25.3, d.f. = 5, *P* < 0.001; Fig. 2), with the highest cumulative survival found in embryos cultured at low temperature with high *p*CO_2_ and the lowest survival of embryos in the elevated temperature, high *p*CO_2_ treatment over the course of the experiment (Sun's score statistic: −1°C, high *p*CO_2_ = −10.53; +2°C, high *p*CO_2_ = 15.69). Overall, survival was higher and very similar among low temperature treatments (Sun's scores statistics: ambient = −7.54, moderate = −9.96 and high = −10.53), while all elevated temperature treatments exhibited higher mortality that increased with *p*CO_2_ levels (Sun's score statistic: ambient = 2.60, moderate = 9.74 and high = 15.69). Over the course of the experiment, survival declined more quickly at elevated temperatures, with cumulative probability of survival at the end of the experiment 68 ± 3 vs. 81 ± 1% at low temperatures (mean ± SEM). Within the elevated temperature treatment, there was an additional decrease in survival associated with *p*CO_2_ treatments (ambient *p*CO_2_, 73 ± 4%; moderate *p*CO_2_, 68 ± 6%; and high *p*CO_2_, 63 ± 3%). Survival data from one replicate bucket from the low temperature and high *p*CO_2_ treatment was omitted from analysis due to unusually high, sudden, unrelated mortality.

### Morphometrics and development

#### Morphometrics

Overall embryo metrics measured at the level of the whole egg did not vary substantially between treatment groups over the course of the experiment. Mean egg diameter was 3.35 ± 0.01 mm [±95% confidence interval (CI), *n* = 449; Supplementary Fig. 1] and mean egg wet mass was 19.62 ± “0.09 mg (±95% CI, *n* = 162; Supplementary Fig. 2). Likewise, total protein per egg did not change over the course of the experiment (1.36 ± 0.1 mg, mean ± 95% CI, *n* = 212, data not shown), which is primarily yolk protein at this stage of early development.

#### Development

Using the estimated lay date of 1 November 2013, embryos entered the experiment at 16–17 days post-fertilization and reached 37–38 days post-fertilization by the end of the experiment (21 days). Over the course of the 3 weeks of experimentation, embryos progressed from the early body patterning of gastrulation (Fig. 1a) to mid-stages of segmentation (∼12–20+ somites), with some embryos extending over 50% of the yolk (Fig. 1b–f). Early in somitogenesis, the first major morphological structures to appear are the optic vesicles (Fig. 1c). Pigments (melanophores), commonly used to identify larval notothenioids to species, began to appear in conjunction with embryonic somitogenesis and growth, and were detected as early as 2 weeks of experimental treatment in some embryos (Fig. 1d) and continued to expand in density and location at 3 weeks as embryos developed (Fig. 1e).

**Figure 3:**
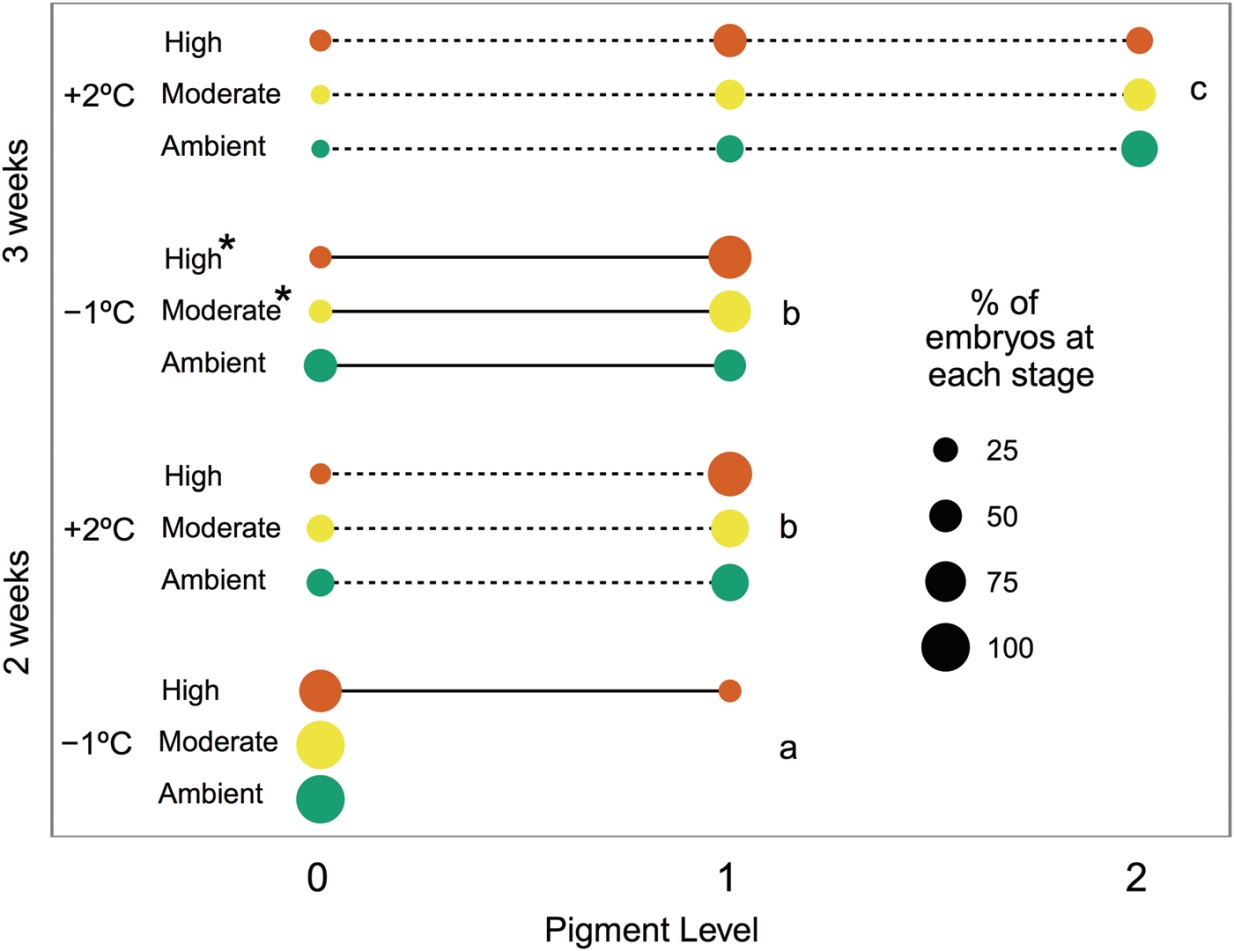
Proportional age class distribution of dragonfish (*G. acuticeps*) embryos by time exposed to either −1 or +2°C and ambient (420 μatm), moderate (650 μatm) or high (1000 μatm) *p*CO_2_ over the course of 3 weeks. Age class is based on amount of pigmentation in unique individuals after 2 and 3 weeks of exposure to experimental conditions. Different letters identify significant differences between temperature treatments. Asterisks represent significant differences between treatments within time points.

After 2 weeks, significant differences in development appeared between embryos held at the two temperatures as determined by levels of pigmentation (Fig. 3). Embryos in the elevated temperature group were significantly more developed than embryos at low temperature at both 2 weeks (pigment level = 1, 7 vs. 73%, χ^2^ = 46.12, *P* < 0.001) and 3 weeks (pigment level = 0, 30 vs. 9.5%; pigment level = 1, 70 vs. 42.5%; and pigment level = 2, 0 vs. 47%, χ^2^ = 46.01, *P* < 0.001). Embryos from the elevated temperature group at 2 weeks were not significantly different from embryos from the low temperature group at 3 weeks, suggesting a developmental acceleration of a week in the embryos acclimated to the elevated temperature (pigment level = 1, 73 vs. 70%, χ^2^ = 0.12, *P* = 0.85). Additionally, embryos in the moderate and high *p*CO_2_ treatments were significantly more advanced than embryos in the ambient *p*CO_2_ treatment at low temperature at 3 weeks (pigment level = 1, 47.8% ambient vs. 79.2% moderate, 82.6% high, χ^2^ = 8.09, *P* = 0.02). Increased *p*CO_2_ may have also caused subtle shifts in development in combination with temperature, because embryos in the high *p*CO_2_ treatment were more advanced overall at 2 weeks (86% of high *p*CO_2_ embryos at level 1 vs. 65% of ambient and 67% of moderate), but fewer high *p*CO_2_ embryos had advanced to the most developed stage at 3 weeks compared with the other groups (32% of high *p*CO_2_ embryos at level 2 vs. 63% of ambient and 50% of moderate).

### Respirometry

**Figure 4:**
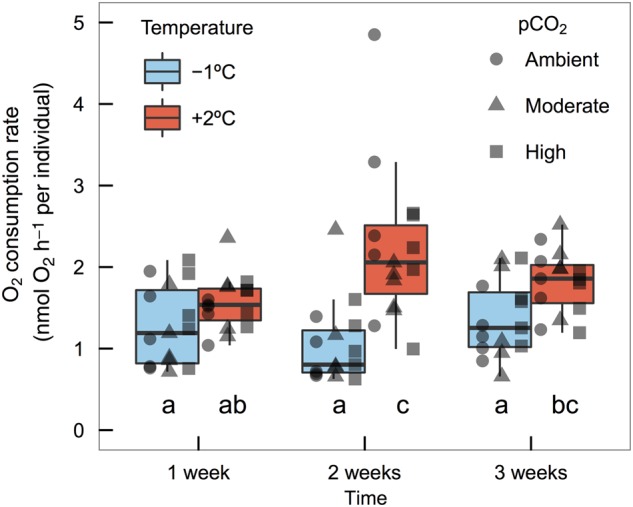
Oxygen consumption (in nanomoles of oxygen per hour per individual) of dragonfish (*G. acuticeps*) embryos exposed to either −1 or +2°C and ambient (420 μatm), moderate (650 μatm) or high (1000 μatm) *p*CO_2_ over the course of 3 weeks. Boxplots represent median, first and third quartiles of time × temperature, which interactively affected rate of O_2_ consumption (*F*_2,72_ = 4.18, *P* = 0.02, *n* = 15). Plotted points are unique values of O_2_ consumption by *p*CO_2_ × temperature × time (*n* = 5). Different letters identify significant differences between groups.

Temperature significantly increased O_2_ consumption rate (*F*_1,72_ = 22.57, *P* = <0.0001) of embryos, with a significant interaction between time and temperature (*F*_2,72_ = 4.18, *P* = 0.02; Fig. 4). Oxygen consumption rate differences at 1 week between low and elevated temperature were small (1.3 ± 0.3 vs. 1.6 ± 0.2 nmol O_2_ h^−1^ per individual, mean ± 95% CI, +23% difference, *n* = 15). At 2 weeks, there was a significant difference in O_2_ consumption rates between the two temperature groups (1.0 ± 0.3 vs. 2.2 ± “0.5 nmol O_2_ h^−1^ per individual, +120% difference), and the significant differences in O_2_ consumption between temperature groups were maintained at 3 weeks (1.4 ± 0.3 vs. 1.8 ± “0.2 nmol O_2_ h^−1^ per individual, +29% increase). No consistent patterns in O_2_ consumption rate by *p*CO_2_ treatment were detected (*F*_2,72_ = 0.19, *P* = 0.83).

### Citrate synthase

**Figure 5:**
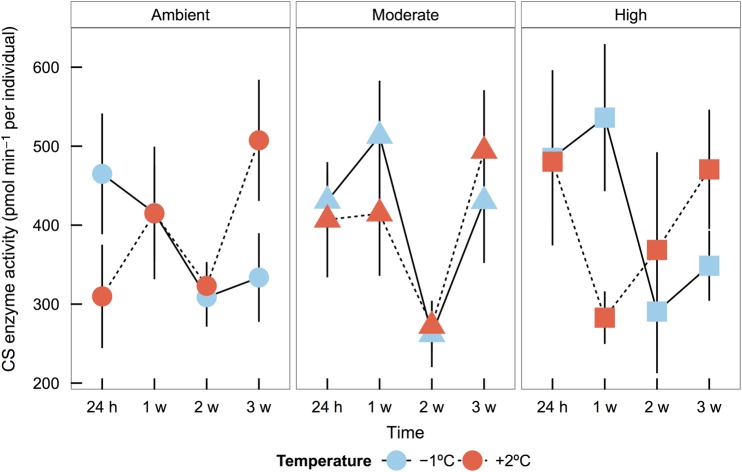
Citrate synthase (CS) enzyme activity (in picomoles per minute per individual) of dragonfish (*G. acuticeps*) embryos exposed to either −1 or +2°C and ambient (420 μatm), moderate (650 μatm) or high (1000 μatm) *p*CO_2_ over the course of 3 weeks. Time, temperature and *p*CO_2_ interactively affected CS activity (*F*_6,179_ = 2.25, *P* = 0.04, *n* = 9). Values are means ± 95% confidence intervals.

Specific activity of CS varied by a complex interaction between time, temperature and *p*CO_2_ treatment during the experiment (*F*_6,179_ = 2.25, *P* = 0.04), with *p*CO_2_ treatment affecting variance differently across time (*L* = 39.67, d.f. = 11, *P* < 0.001). In order to provide the best reflection of the three-way statistical interaction, results are presented (Fig. 5) and discussed by specific *p*CO_2_ treatment groups.

#### Ambient *p*CO_2_ treatment

Citrate synthase activity generally decreased over time in the low temperature group, while CS activity increased over time at elevated temperature, with differences in mean CS activity between the start (24 h) and end (3 weeks) of the experiment. Specifically, at 24 h the CS activity was 33% less at the elevated temperature, but at 3 weeks CS activity was 52% higher in the elevated vs. low temperature groups.

#### Moderate *p*CO_2_ treatment

Both temperature groups exhibited very similar CS activity trends over time. At 24 h and 1 week, CS activities remained elevated, before declining at 2 weeks to the lowest observed activity levels in the experiment. At 3 weeks, CS activity levels increased to levels similar to those of the rest of the elevated temperature treatments, with activity 15% greater in the +2°C group.

#### High *p*CO_2_ treatment

Citrate synthase activity patterns varied over time depending on temperature. Starting from relatively high activity at 24 h in both groups, at elevated temperature CS activity sharply declined at 1 week, followed by subsequent increases at both 2 and 3 weeks. At low temperature, activity remained high at 1 week before decreasing to low activity observed at 2 and 3 weeks.

Comparing CS activity values at 3 weeks by temperature and *p*CO_2_ treatment, rates were greatest at the elevated temperatures (mean ± 95% CI: ambient *p*CO_2_, 507 ± 77 pmol min^−1^ per individual; moderate *p*CO_2_, 494 ± 77 pmol min^−1^ per individual; and high *p*CO_2_, 471 ± 76 pmol min^−1^ per individual), followed by the moderate *p*CO_2_ treatment in the low temperature group (430 ± 78 pmol min^−1^ per individual). Lowest CS activity at 3 weeks occurred in the ambient and high *p*CO_2_ treatments in the low temperature group (ambient *p*CO_2_, 334 ± 56 “pmol min^−1^ per individual; and high *p*CO_2_, 349 ± 44 “pmol min^−1^ per individual).

### Osmolality

**Figure 6:**
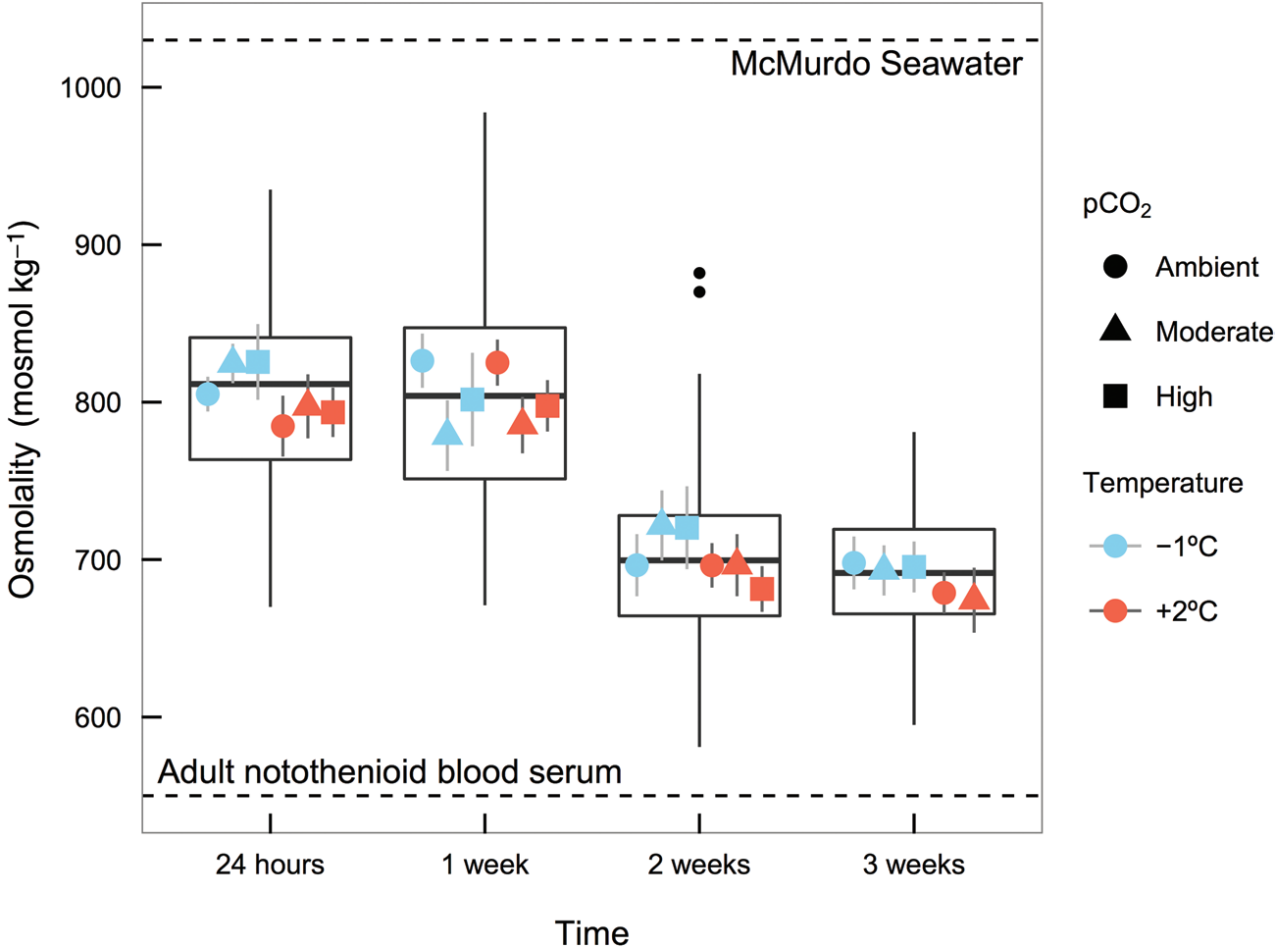
Whole-egg osmolality (in milliosmoles per kilogram) of dragonfish (*G. acuticeps*) embryos exposed to either −1 or +2°C and ambient (420 μatm), moderate (650 μatm) or high (1000 μatm) *p*CO_2_ over the course of 3 weeks. Values of local seawater and adult fish serum osmolalities (Cheng and Detrich, 2007) are presented as dashed horizontal lines. Boxplots represent median, first and third quartile values by time, and points represent temperature × *p*CO_2_ (means ± SEM).

Dragonfish embryos are hyposmotic to their seawater environment (1030 mosmol kg^−1^) but have a greater osmolality than the blood serum of adult notothenioids (550 mosmol kg^−1^; Cheng and Detrich, 2007). Osmolality was significantly greater at 24 h and 1 week (805 ± 14 and 802 ± 17 mosmol kg^−1^, respectively) than at 2 and 3 weeks (702 ± 16 and 689 ± 14 mosmol kg^−1^, respectively) across all temperature and *p*CO_2_ treatments (±95% CI, *n* = 54 for 24 h, 1 and 2 week; or *n* = 40 for 3 weeks; *F*_3,157_ = 62.65, *P* < 0.001; Fig. 6). Overall, osmolality declined by 13% between the first week of the experiment and the later 2 weeks. Embryos in the elevated temperature group generally exhibited a slightly lower osmolality compared with the low temperature group (*F*_1,157_ = 4.07, *P* = 0.045), but we did not detect differences in mean values by *p*CO_2_ level (*F*_2,157_ = 0.13, *P* = 0.87).

## Discussion

Future ocean warming and acidification have the potential to alter the physiology and development of Antarctic fishes significantly. Our results from dragonfish embryos during 3 weeks of exposure to near-future ocean conditions provide evidence that temperature will probably be the main driver of change, but increases in *p*CO_2_ will also alter embryonic physiology, with responses dependent on water temperature (i.e. synergistic interaction of temperature and *p*CO_2_). A moderate increase in temperature (+3°C above ambient temperatures) increased mortality, rate of development, whole-organism respiration rate and cellular aerobic capacity. Exposure to increased *p*CO_2_ (650 and 1000 μatm) and increased temperature led to a synergistic increase in mortality as *p*CO_2_ level increased. Temperature modulated whole-organism metabolic rate, while development and cellular metabolic rate were sensitive to increased *p*CO_2_ depending on time and temperature. Interindividual variability in response to temperature varied by time and *p*CO_2_ treatment, highlighting the potential for within-population variability in response to future ocean change.

Dragonfish embryonic survival was negatively impacted by increased temperature, and embryos exhibited a synergistic increase in mortality when elevated temperature was coupled with increased *p*CO_2_ over the course of the 3 week experiment. In fact, the lowest mortality was experienced by the embryos within the low temperature, high *p*CO_2_ treatment and the greatest mortality was experienced by embryos held for 3 weeks at elevated temperature, high *p*CO_2_. Our findings are consistent with several other studies showing no changes in survival of fish embryos during exposure to future ocean acidification conditions (Munday *et al*., 2009b; Franke and Clemmesen, 2011; Frommel *et al*., 2013). However, given the slow ontogenetic development of *G. acuticeps*, our experimental period may have been insufficient to detect negative effects of increased *p*CO_2_ alone documented in other fish species, such as abnormal development (Baumann *et al*., 2011, Forsgren *et al*., 2013). Very few studies, however, have exposed fish embryos to increased temperature and *p*CO_2_ concurrently. In flatfish, increased temperature caused a decrease in hatching success, with a slight, but non-significant, decrease in hatching when both temperature and *p*CO_2_ were elevated (Pimentel *et al*., 2014b). In contrast, while the sensitivity of marine invertebrate larvae to ocean acidification depends largely on their calcification strategy, most studies on invertebrates have found negative, additive effects of temperature and *p*CO_2_ on survival (Byrne and Przeslawski, 2013; Harvey *et al*., 2013; Kroeker *et al*., 2013). Similar to our findings, there was a negative synergistic effect of increased *p*CO_2_ and projected summer ocean warming temperatures on European squid embryonic survival (Rosa *et al*., 2014). Our findings provide strong evidence that studies focusing on single stressors alone may not be sufficient to predict the effects of concurrent stressors on survival in the early development of fishes, because the negative effects of increased *p*CO_2_ may manifest only at increased temperature.

Dragonfish embryos exposed to warmer, more acidic waters for 21 days developed faster than control embryos at low temperature and ambient *p*CO_2_. Individually, increased temperature and *p*CO_2_ had significantly positive effects on developmental rate. After 2 weeks in the experiment, embryos in the elevated temperature group were ∼1 week ahead developmentally, and the separation in developmental timing increased further after 3 weeks. While we predictably found that temperature increased embryonic development, altered development due to increased *p*CO_2_ was unexpected. At low temperature, both moderate and high *p*CO_2_ exposure significantly increased the proportion of embryos progressing to the first pigment stage at 3 weeks compared with embryos at control *p*CO_2_. When embryos experienced high *p*CO_2_ and increased temperature, we observed trends for high *p*CO_2_ additively to increase the proportion of more developed embryos at 2 weeks. However, at 3 weeks, high *p*CO_2_ appeared antagonistically to slow development when compared with embryos that developed in the presence of ambient and moderate *p*CO_2_. From a physiological perspective, it can be difficult to compare fish embryos that have the same age but develop at different temperatures and to separate the effects of developmental stage from those of temperature on physiological performance (Geffen and Nash, 2012). Taking into consideration the ∼1 week acceleration in development of the elevated temperature groups, it appears that differences in both developmental stage and environmental conditions are driving differences in embryonic physiology (Supplementary Table 1). Given that developmental staging was conducted weekly in the present experiment, a more fine-scale characterization of developmental timing in future ocean conditions is necessary so that embryos can be compared at similar developmental stages.

In other ocean acidification studies on marine embryos, exposure to high *p*CO_2_ resulted in no change [orange clownfish (*Amphiprion percula*), Munday *et al*., 2009b], temporary delay [medaka (*Oryzias latipes*), Tseng *et al*., 2013] or persistent delay [European squid (*Loligo vulgaris*), Rosa *et al*., 2014] in development until hatching. An increased rate of development in the presence of increased *p*CO_2_ may be a side-effect of increased metabolism to cope with stress or a more complex response interacting with developmental processes (i.e. altered cell signalling or gene expression). In our study, the changing effects of high *p*CO_2_ at high temperature on developmental rate over the course of the 3 week acclimation period might be a result of differences in the *p*CO_2_ sensitivity of stage-specific processes. The cellular mechanisms underlying why *p*CO_2_ might advance development during some periods but impair development at other periods require further investigation in order to understand the effects of elevated *p*CO_2_ on long-term development and hatching in dragonfish embryos.

Although our study monitored developmental changes over a relatively short period of embryonic development, observed accelerations in development from warming or acidification have the potential substantially to shorten the embryonic duration of dragonfish (normally ∼310 days). In nature, dragonfish embryos were observed to be pigmented and encircling ∼60% of their yolk at ∼120 days post-fertilization during the end of January (Evans *et al*., 2005). Compared with the pigmentation observed in the present study, the most developed embryos in our elevated temperature group appear to be ∼2 months ahead of normal development. Estimating the time to hatch using the growing degree days (Neuheimer and Taggart, 2007) approach as ∼992 (using an average environmental temperature of −1.8°C and a threshold temperature *T*_0_ of −5°C from Cziko *et al*., 2006), embryos in seawater at +2°C could hatch as early as March, during the end of austral summer. Evans *et al*. (2005) witnessed early hatching in March, April and May (majority hatching time, estimated 1119 growing degree days for 63 days at −1.6°C, 146 days at 1.2°C) for an egg clutch transferred to +1 to +1.4°C in New Zealand in aquarium conditions, which supports our speculation of even earlier hatching when developing at +2°C. Hatching during the beginning of winter, a period when some adult notothenioids undergo metabolic hibernation to survive limited food resources (Campbell *et al*., 2008), could have negative consequences for successful larval development if no food resources are available during this light-limited season of low productivity.

Oxygen consumption rates were thermally sensitive, but not sensitive to *p*CO_2_ exposure. Initial small increases in O_2_ consumption at higher temperatures after 1 week became significantly more pronounced at 2 and 3 weeks, when developmental differences also emerged between embryos held in different temperature treatments. Our study provides the first data available for Antarctic fishes on the effects of temperature on embryonic development, contributes to the well-established body of literature in fishes from other ecosystems (Pepin, 1991) and builds upon previous measurements of embryonic O_2_ consumption in *G. acuticeps* in ambient conditions (Evans *et al*., 2006). There was no effect of *p*CO_2_ treatment on O_2_ consumption at either temperature at any time during the 3 week experiment, suggesting that dragonfish embryos do not alter their whole-organism metabolic rate with increasing *p*CO_2_ during early development. Considering O_2_ consumption in embryogenesis as a proxy for energetic demand for both basal maintenance and development, we expected that increased *p*CO_2_ could increase O_2_ consumption due to increased energy demand for acid–base regulation during acidification (Pörtner, 2012; Sokolova *et al*., 2012). As O_2_ consumption did not change in our experiment in the ocean acidification treatments, the levels of *p*CO_2_ may not have significantly altered extra- and intracellular pH levels of the embryos enough to incur additional metabolic costs (Melzner *et al*., 2009). Alternatively, individual embryos, known to exhibit intraspecific variation in metabolic rates in normal conditions (Bang *et al*., 2004), may exhibit variable metabolic responses to *p*CO_2_ that were not fully captured when pooled as a group. While we are not aware of any other studies that have measured O_2_ consumption rates of fish embryos according to near-future ocean acidification predictions, our findings suggest that fish may differ from many marine invertebrate embryos in how they respond to *p*CO_2_. Metabolic suppression after high *p*CO_2_ exposure was observed in late-stage porcelain crab (*Petrolisthes cinctipes*) embryos (Carter *et al*., 2013) and squid embryos when also exposed to increased temperature (Rosa *et al*., 2014), but not in the Norway lobster (*Nephrops norvegicus*; Styf *et al*., 2013). However, the effects of embryonic exposure may have carry-over effects to other life stages because O_2_ consumption and metabolic activities are lower in recently hatched dolphinfish larvae after embryonic exposure to high *p*CO_2_ (Pimentel *et al*., 2014a). Changes in O_2_ consumption during late-stage embryonic development and post-hatching in the presence of increased *p*CO_2_ in other marine ectotherms may reflect a change in physiological mechanisms (i.e. gill and cardiac development, embryo movement, hatching) available for adjusting metabolism at later ontogenic stages.

In comparison to whole-organism aerobic metabolism, CS activity, an index of cellular aerobic capacity, was seen to be dependent on a complex interaction of temperature, *p*CO_2_ and time. After 3 weeks, embryos in the elevated temperature treatment, across all *p*CO_2_ levels, exhibited the highest CS activity as well as having the most developed embryos. Citrate synthase activity over time was very similar between temperature treatments at moderate *p*CO_2_ exposure, including elevated rates after 3 weeks; whereas embryos exposed to ambient or high *p*CO_2_ altered CS activity in a different manner at low vs. high temperature throughout the duration of the experiment. These findings suggest that dragonfish embryos may respond to changes in both ocean warming and acidification by altering cellular aerobic metabolism to maintain energy supply or compensate for altered cellular conditions. When exposed to increased *p*CO_2_, medaka fish embryos reduced gene expression of CS and other metabolic enzymes and experienced a transient delay in development during a period of hypothesized insufficient acid–base regulation capacity (Tseng *et al*., 2013). In an adult Antarctic notothenioid fish (*Notothenia rossii*), increases in intracellular bicarbonate to compensate for hypercapnia (Strobel *et al*., 2012) may competitively inhibit CS enzyme function and reduce mitochondrial capacities (Strobel *et al*., 2013a), leading to increased CS activity in highly aerobic tissues, such as red muscle (Strobel *et al*., 2013b). Although adult acid–base regulation capacity has not yet developed, CS activity in dragonfish embryos may be reflective of similar changes in intra- and extracellular processes that may also reflect altered gene expression. Similar to whole-organism metabolic rate, CS activity was greater overall in embryos exposed to elevated temperatures at the end of the experiment; however, the patterns we observed over time in CS activity suggest a much more dynamic role of cellular aerobic activity during early development in dragonfish compared with patterns at the whole-organism level. Our findings suggest that overall cellular aerobic capacity may respond to changes in *p*CO_2_ in ways that alter net energy generation within the embryo, showing the importance of integration across physiological levels to obtain a fuller, albeit more complex, understanding of metabolic responses to changing environments.

Dragonfish embryos in our study showed vulnerability to changing environmental conditions during a relatively small portion of their life cycle. It is important to note that we assessed one clutch of eggs from two presumed parental sources, and therefore future work is needed to test whether genetic variability and parental effects alter the response to temperature or acidification, as observed in other studies of marine organisms that specifically tested for clutch effects (Chan *et al*., 2011; Foo *et al*., 2012; Carter *et al*., 2013; Hurst *et al*., 2013). While more comprehensive and longer-term studies are needed, the lower survival of embryos reared at +2°C, high *p*CO_2_ may suggest the potential for reduced hatching success with ocean warming and acidification. Beyond reduced numbers for recruitment, the detected increases in developmental rate and metabolism during warming and acidification also have the potential to impact the successful transition to larvae by leading to early hatching. At present, dragonfish hatch and develop in the spring and summer, an annually productive time period when most other notothenioid fish also hatch due to food availability and beneficial growth conditions (Koubbi *et al*., 2009). As the successful transition from larval to juvenile life stages requires a high energetic demand to support successful growth, development and survival (Post and Parkinson, 2001), seasonal mismatch with prey species, such as copepods, could severely reduce larval recruitment (Beaugrand *et al*., 2003). Future ocean conditions will also affect the environment of marine fishes throughout their entire life history, and previous research has demonstrated that adult Antarctic fishes also experience increased metabolic demands in future climate change (Strobel *et al*., 2012, 2013a,b; Enzor *et al*., 2013). As parental dragonfish devote energy into 10 months of nest guarding during embryonic development (Evans *et al*., 2005), parental care behaviours may be altered with ocean climate change, as observed in three-spined stickleback (Hopkins *et al*., 2010), as the demand for energy and therefore foraging increase. Additionally, Antarctic fishes, like other slow-growing but long-lived species, take many years to reach reproductive maturity (Mesa and Vacchi, 2001), reproduce only annually (Kock and Kellermann, 1991) and depend on food resources that may also be affected by future ocean conditions (Kawaguchi *et al*., 2011). By confirming the vulnerability of the early life stages of Antarctic fish to climate change, we now have another piece in the complex puzzle in predicting how diverse ecosystems and organisms will cope with warmer, more acidic oceans.

## Supplementary material

Supplementary material is available at *Conservation Physiology* online.

## Funding

This work was supported by the National Science Foundation [NSF ANT-1142122 to A.E.T.], an Achievement Rewards for College Scientists (ARCS) Foundation award to E.E.F., a Council on Ocean Affairs, Science and Technology (COAST) student award to E.E.F. and a San Francisco State University Biology Department scholarship to E.E.F.

## Supplementary Material

Supplementary Data
