# Supplementary material for: Ocean acidification exerts negative effects during warming conditions in a developing Antarctic fish
Source: Conserv Physiol. 2015 Jul 27;3(1):cov033. doi: 10.1093/conphys/cov033 (PMC4778439; doi:10.1093/conphys/cov033)

Supplementary Figure Legends

Supplementary Figure 1. Frequency distribution of egg diameters of experimental dragonfish (*Gymnodraco acuticeps*) embryos.


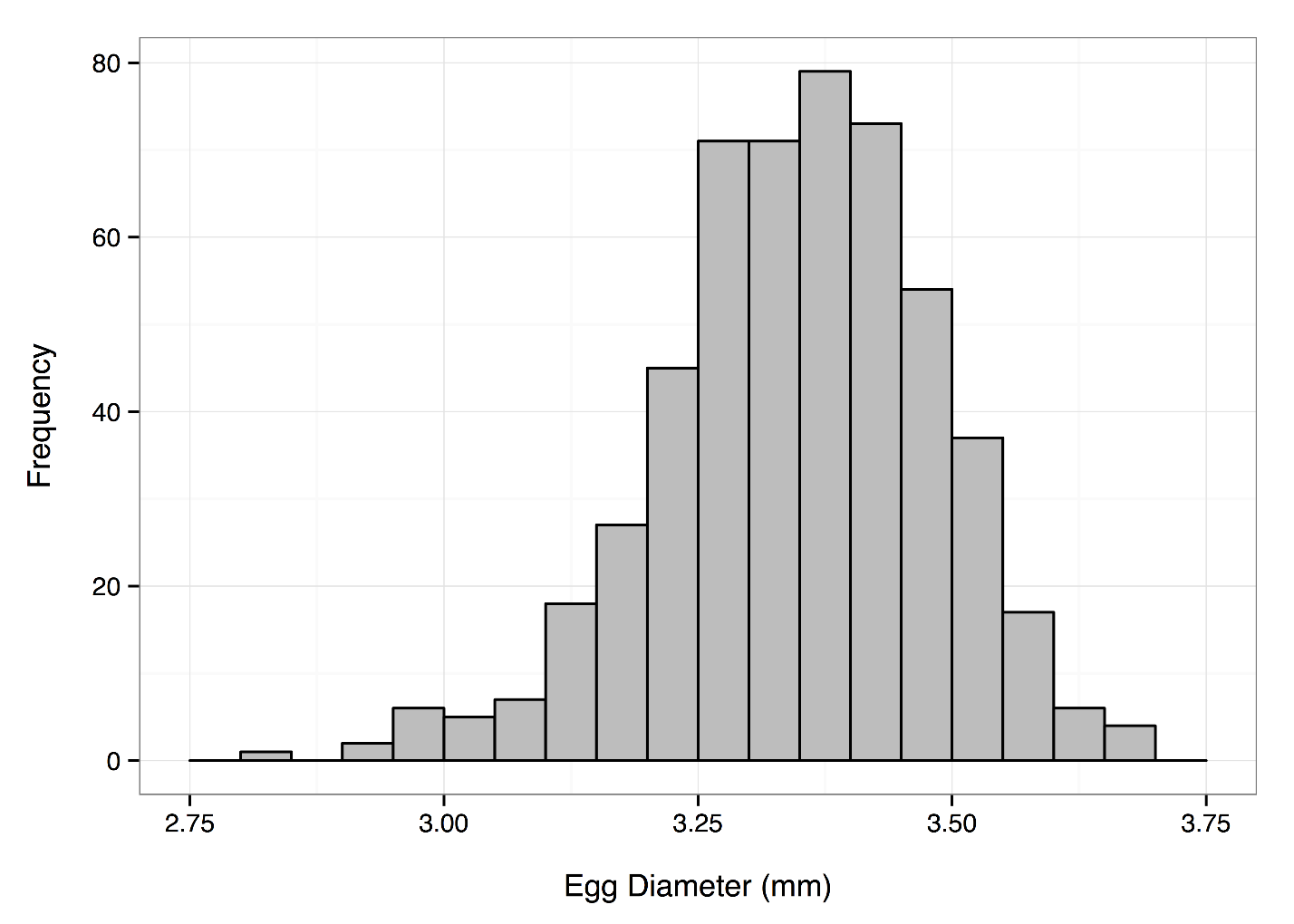


Supplementary Figure 2. Frequency distribution of egg wet mass of experimental dragonfish (*Gymnodraco acuticeps*) embryos.


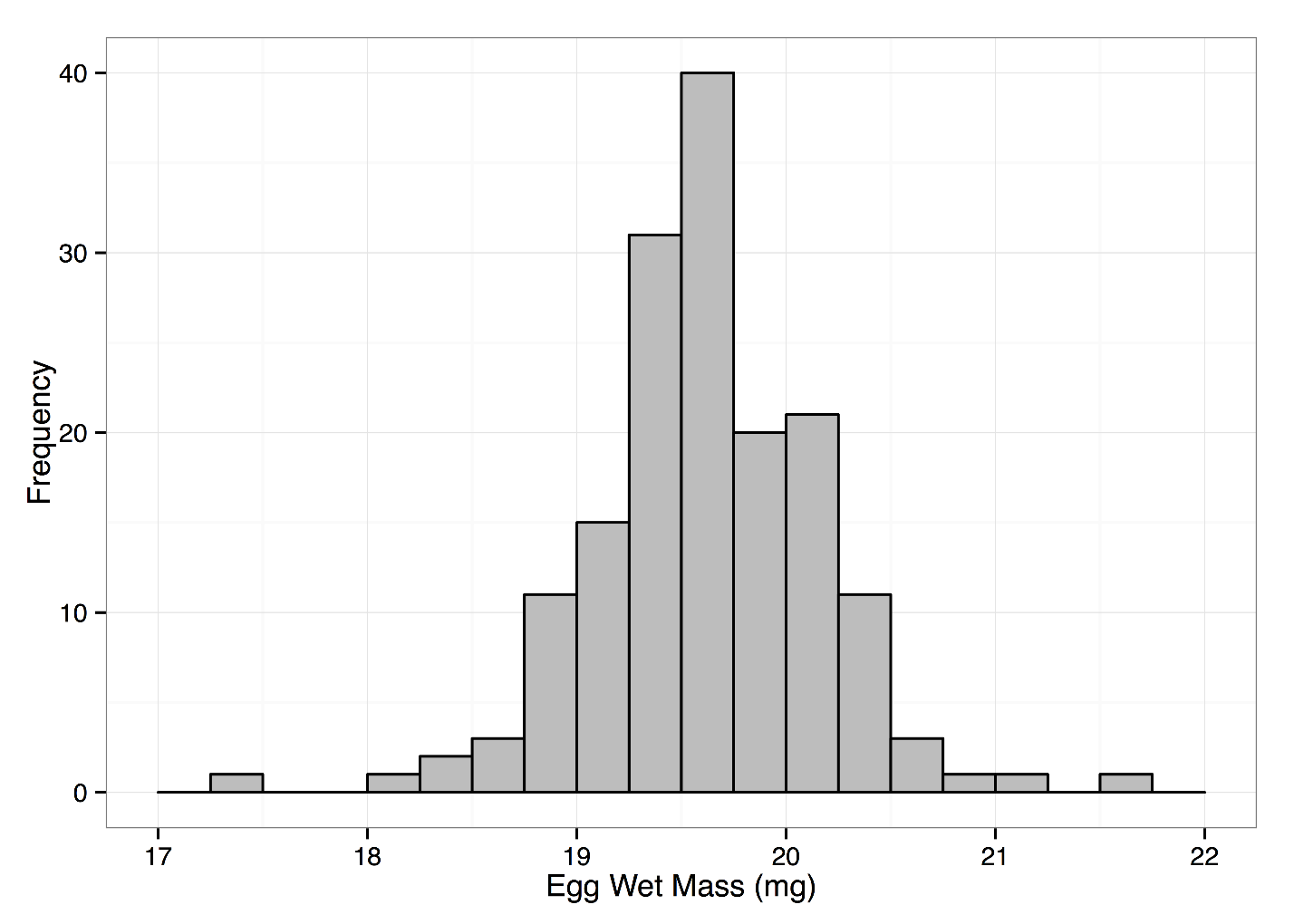

Supplement: Supplementary Data [file cov033supp.zip › cov033supp_fig1.docx]
