# Supplementary material for: Ocean acidification exerts negative effects during warming conditions in a developing Antarctic fish
Source: Conserv Physiol. 2015 Jul 27;3(1):cov033. doi: 10.1093/conphys/cov033 (PMC4778439; doi:10.1093/conphys/cov033)
Supplement: Supplementary Data [file cov033supp.zip › cov033supp_table1.docx]

Supplementary Table 1. Comparison of dragonfish (*G. acuticeps*) embryos after 3 weeks at low temperature (-1°C) vs. 2 weeks at elevated temperature (2°C) by *p*CO_2_ level (ambient [400 μatms], moderate [650 μatms], or high [1000 μatms]). All values are means ± standard error, except for development stages values which represent % of total embryos at each stage. Numbers for each treatment are the following: survival (n=3 [n=2 for low temperature, high *p*CO_2_]), respirometry (n=5), development (n=21-23), and citrate synthase and osmolality (n=9).
